# Degradation of Microplastics by Zinc Oxide Nanoparticles Synthesized Using Piper longum Leaf Extract

**DOI:** 10.7759/cureus.69876

**Published:** 2024-09-21

**Authors:** Nazleen V Vas, Shantha K Sundari, Sivakamavalli Jeyachandran

**Affiliations:** 1 Orthodontics and Dentofacial Orthopaedics, Saveetha Dental College and Hospitals, Saveetha Institute of Medical and Technical Sciences, Chennai, IND

**Keywords:** degradation, microplastics, piper longum, plastic waste, zinc oxide nanoparticles (zno)

## Abstract

*Background*: The environmental hazards posed by microplastics have drawn considerable concern due to their buildup in ecosystems. Microplastics accumulate in human saliva, skin, and hair. Developing effective technology for managing and degrading microplastics remains a substantial challenge. In a concerted attempt to save the ecology, this study explores the photocatalytic breakdown of common microplastics like polystyrene (PS) microspheres and polyethylene (PE) using green-synthesized zinc oxide nanoparticles (ZnO NPs) under UV light exposure.

*Aim*: To synthesize and characterize zinc oxide nanoparticles prepared using the extract of the leaves of *Piper longum *and qualitatively assess the photocatalytic degradation potential of the nanoparticles under light microscopy.

*Material and Methods*: A fresh extract of *P. longum* leaves was used as a reducing agent to synthesize zinc oxide nanoparticles. Fourier transform infrared (FTIR) analysis, X-ray diffraction (XRD) analysis, UV-Vis spectra analysis, and scanning electron microscopy (SEM) analysis were performed to characterize the nanoparticles. Microplastics were isolated from the saliva of 50 healthy patients and were purified and filtered. In a six-well microtiter plate, 0.5 μg of varying concentrations of nanoparticles were added. After fixing with 15% formaldehyde, microplastics were subjected to UV irradiation for 2 hours with different concentrations of ZnO nanoparticles (25, 50, 75, and 100 µg). Custom photoreactors activated the photocatalysts to degrade the microplastic pollutants. The six-well microtiter plate was viewed under 40x magnification in a light microscope to observe microplastic morphology after 24 hours of degradation.

*Results*: The FTIR spectrum showed distinct peaks at 890.51 cm⁻¹, indicating the involvement of C-N in-plane vibrations of amino acids. XRD analysis revealed three distinct diffraction peaks at 31.68°, 34.39°, and 36.33°, corresponding to the hexagonal wurtzite structure of ZnO nanoparticles. The synthesized ZnO nanoparticles ranged from 50 to 90 nm in size, viewed at 100x magnification on SEM. The highest degradation of microplastics was observed at a ZnO NP concentration of 100 µL, with the ZnO NPs 50-90 nm in size.

*Conclusion*: Zinc oxide nanoparticles synthesized using *Piper longum* leaf extract effectively degrade microplastics, with the highest degradation observed at a 100 µL concentration of ZnO nanoparticles and optimal degradation occurring at a concentration of 75 µL.

## Introduction

Plastics are favored for their lightweight, affordability, and durability; however, their disposal presents a growing challenge. Over 8.3 billion metric tons of plastic enter water bodies annually, with projections indicating that plastic pollution will exceed that of oceanic fish shortly [1]. Plastic typically breaks down into smaller particles through a combination of factors such as sunlight exposure, physical wear, and microbial action, resulting in the formation of microplastics (5 mm and less) and nano-plastics (1 mm and less) [2]. Despite increasing efforts in recycling, a significant portion of plastics remains difficult or uneconomical to recover [3]. Common plastics like polyethylene (PE) and polystyrene (PS) are widely used in various applications such as building materials, packaging, and food containers [4]. However, they can enter water environments through improper disposal, wastewater treatment, and microbial breakdown [5]. Due to their persistent characteristics, PE and PS pose risks to both human health and aquatic life. Their presence in aquatic ecosystems poses significant threats to marine and terrestrial organisms through various pathways in the food chain. The complexity and severity of plastic waste issues underscore the urgent need for novel approaches to polymer decomposition.

The discovery of nanoparticles and nanotechnology has far-reaching implications in almost every industry, be it engineering and electronics, agriculture, or the medical field at large. Within the dental field, the effects of development in nanotechnology have created ripples through its various branches. Due to their special qualities and their uses in catalysis, biological tagging, optoelectronics, photonics, and pharmaceuticals, metal nanoparticles have drawn a lot of interest recently. An n-type semiconducting metal oxide is zinc oxide (ZnO). The existence of various morphologies of zinc oxide nanoparticles (ZnO NPs) like nanowire, nanorod, nanobelt, nanoflake, and nanoflower have been reported. ZnO NPs have been applied to various biomedical therapies including anti-diabetic, antifungal, antibacterial, and anti-cancer, for drug transport purposes as well as in other fields like agriculture. ZnO NPs have been shown in studies to have antimicrobial activity against *Staphylococcus epidermidis* and *Escherichia coli* [6-8].

In orthodontics, this property has been exploited by the incorporation of ZnO nanoparticles in Transbond XT adhesive primer to achieve antimicrobial activity against *Streptococcus mutans* [9]. Similarly, an orthodontic composite with ZnO nanoparticles and chitosan nanoparticles in 10% weight concentration was found to have antimicrobial activity against *Lactobacillus acidophilus*. Another study that incorporated ZnO nanoparticles into a composite demonstrated antimicrobial activity against *Lactobacillus*, *Streptococcus mutans*, and *Candida albicans* at concentrations of ZnO less than 5% [10]. In another study, brackets coated with zinc/silver oxide nanoparticles showed enhanced antibacterial effects against *Lactobacillus acidophilus* and *S. mutans* [11]. Orthodontic bands with a nano-ZnO/Ag coating were found to have antibacterial qualities that prevent oral infections. Of all the nanoparticles, nano-Ag's antibacterial activity and nano-ZnO's biocompatibility were reportedly the best [12]. The coating of stainless steel (SS) wires with ZnO nanoparticles was found to reduce abrasion between the bracket and 0.019x 0.025 inch and 0.016x0.022 inch SS wires [13]. ZnO nanoparticles were applied to break down low-density polyethylene film (LDPE) microplastic pieces in water utilizing visible light-excited hetero-ZnO photocatalysts. This was another impressive application of ZnO nanoparticles [14].

Among the various methods employed to synthesize ZnO nanoparticles, biological synthesis achieves both the fabrication of a benign nanostructure and a reduction in the hazardous substances generated in the process. ZnO NPs have previously been green-synthesized using *Trifolium pratense* flower extract, fruit extract of *Rosa canina*, leaf extract of aloe vera. A member of the Piperaceae family, *Piper longum* L., often known as Pippali or Indian long pepper, has long been used to treat a variety of illnesses, including gonorrhea, respiratory tract infections, menstrual discomfort, arthritic disorders, and tuberculosis. *P. longum* is reported to include the three main active ingredients: piperine, piperlongumine, and piplartine. In addition to the active ingredients, which include various alkaloids, piperine, piperidine, tannins, terpenines, sesamin, and isobutyl dienamide, dihydrostigmasterol also contains 4-5% essential oil extracted from catkins. Rich in a variety of bioactive components, the plant extract exhibits potential as an antioxidant, anti-inflammatory, and anti-cancer agent. *P. longum* fruit extracts have previously been used to synthesize silver, copper, and palladium nanoparticles [15-17]. The synthesized copper nanoparticles displayed antimicrobial activity against *Bacillus subtilis*. Thus, the scope of this study is to synthesize and characterize zinc oxide nanoparticles using the extract of the leaves of *P. longum*, and qualitatively assess the photocatalytic degradation potential of the nanoparticles under light microscopy.

## Materials and methods

Preparation of plant extract

Before usage, the glassware and the mortar and pestle were autoclaved. The leaves of a *P. longum* plant were procured from a Chennai-based nursery. Before grinding, the mortar and pestle were pre-chilled in a 95% ethanol solution at -20°C to minimize frozen tissue thawing. Fresh *P. longum* leaves were thoroughly cleaned with tap water, followed by distilled water, to remove contaminants. Using a mortar and pestle, the shade-dried leaves were finely powdered. Then, for 10 minutes at 70°C, 5 g of the dried leaf powder was boiled in 100 ml of sterile, filtered distilled water. After that, the mixture was filtered using No. 1 Whatman filter paper. Consequently, *P. longum* leaf extract, produced as a pale-yellow solution, was stored at 4°C for further use.

*P. longum* extract-mediated synthesis of ZnO NPs

**Figure 1:**
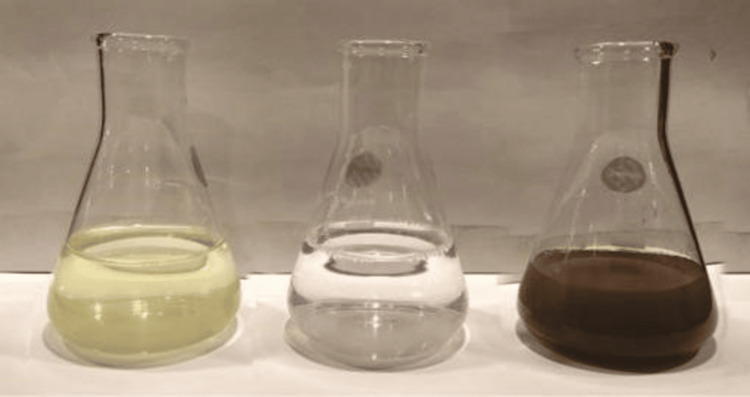
The solution changed color from yellow to brown at the synthesis phase of the nanoparticle

*P. longum-*synthesized ZnO nanoparticles (PL ZnO NPs) were synthesized by soaking 10 mL of *P. longum* aqueous leaf extract in 90 mL of a 1 mM zinc oxide solution. To prevent ZnO photoactivation under static conditions, the suspension was incubated at 37°C in a dark environment. The reaction mixture was centrifuged for 2 hours at 100 rpm. As shown in Figure 1, a yellowish-brown coloring was seen during the synthesis.

Characterization

Fourier Transform Infrared (FTIR)

To identify the functional groups that cause the reduction of Zn ion, FTIR spectroscopy was utilized. IR spectra were acquired between the wavelengths of 4000 and 400 cm^-1^.

X-Ray Diffraction (XRD)

XRD lattice was recorded using JEOL model JPX-8030 instrument (JEOL, Akishima, Tokyo, Japan), which is a computational XRD system, with CuK(alpha) radiation at a range of 20 Å at 40 kV. Syn master 7935 software was used to identify the peak table and for the identification of the XRD peak. Using Bragg’s law, the size of the ZnO NPs was determined from XRD peak positions.

UV-Vis Spectra Analysis

An UV-Vis spectrophotometer (Shimadzu, Kyoto, Japan) was utilized to analyze the produced ZnO NPs in the wavelength range from 450 to 490 cm^-1^ [18].

Scanning Electron Microscopy (SEM)

SEM was performed utilizing a JEOL SEM device (JEOL, Akishima, Tokyo, Japan). Sample films were coated on a carbon grid, excess solution was blotted, and the films were dried for 5 minutes under a mercury lamp.

Collection of oral samples for microplastics isolation

Saliva was collected from 50 healthy subjects. Out of the collected samples, 100 ml of saliva was then processed with hydrogen peroxide and NaCl to digest the other organic matter and to obtain a clarified oral suspension with microplastics. Microplastics were isolated using a 45 mm filter membrane used to filter the isolates from the oral suspension.

Microplastic degradation 

In a six-well microtiter plate, 0.5 μg of varying concentrations of nanoparticles was added. A 15% formaldehyde solution was used to fix the microplastics. The degradation of microplastics involved the utilization of microplastics extracted from saliva as described above, and an evaluation of microplastics was made. To assess the efficacy of catalysts in microplastic degradation, the reduction in microplastics was visualized both before and after subjecting them to a UV irradiation treatment for 2 hours. This treatment involved varying concentrations of PL ZnO NPs (25, 50, 75, and 100 µg) as a catalyst. Custom photoreactors were employed for activating the photocatalysts to facilitate the breakdown of microplastic pollutants.

Microscopic analysis

The six-well microtiter plate was the viewed under 40x magnification under a light microscope to view the morphology of the microplastics after 24 hours of degradation.

## Results

UV-Vis spectroscopy

The absorption spectra of the synthesized *P. longum* ZnO NPs were examined using a UV-Vis spectrophotometer. The solution turned from a pale yellowish-brown to a dark brown tint. The UV-Vis spectrophotometer was used to analyze the fluid that had changed color. The peaks were in the range of wavelength from 300 to 600 nm.

Fourier transform infrared spectroscopy

**Figure 2:**
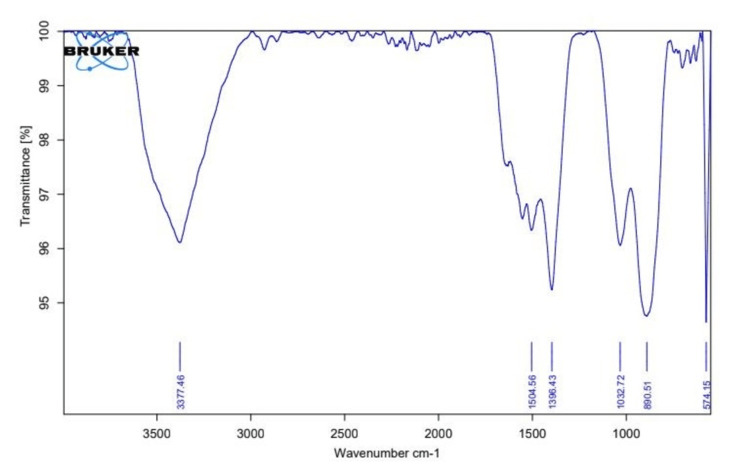
FTIR spectrum shows distinct peaks at 890.51 cm−1, which represent the involvement of C-N in-plane vibrations of amino acids.

The FTIR analysis of the green-synthesized ZnO NPs revealed a vast number of functional groups. N-H groups of proteins were present in bands identified at 1504.56, 1396.43, 1032.72, 890.51, and 574.15 cm^-1^. Numerous smaller peaks between 3000 and 2000 cm^-1^ indicated that the minimal C-H functional group and numerous other bands followed the same pattern as the plant extract. Other peaks in the spectra indicated the existence of numerous functional groups, such as the high peak at 3377.46 cm^-1^, which pointed to the O-H functional group. The presence of different groups suggests that certain phytochemicals have moved from plant extract to zinc oxide nanoparticles (ZnO NPs), where they have likely served as the capping and reducing agents during biosynthesis. The possible functional groups of phytochemicals in plant extract involved in nanoparticle synthesis were identified by FTIR analysis depicted in Figure 2.

X-ray diffraction

**Figure 3:**
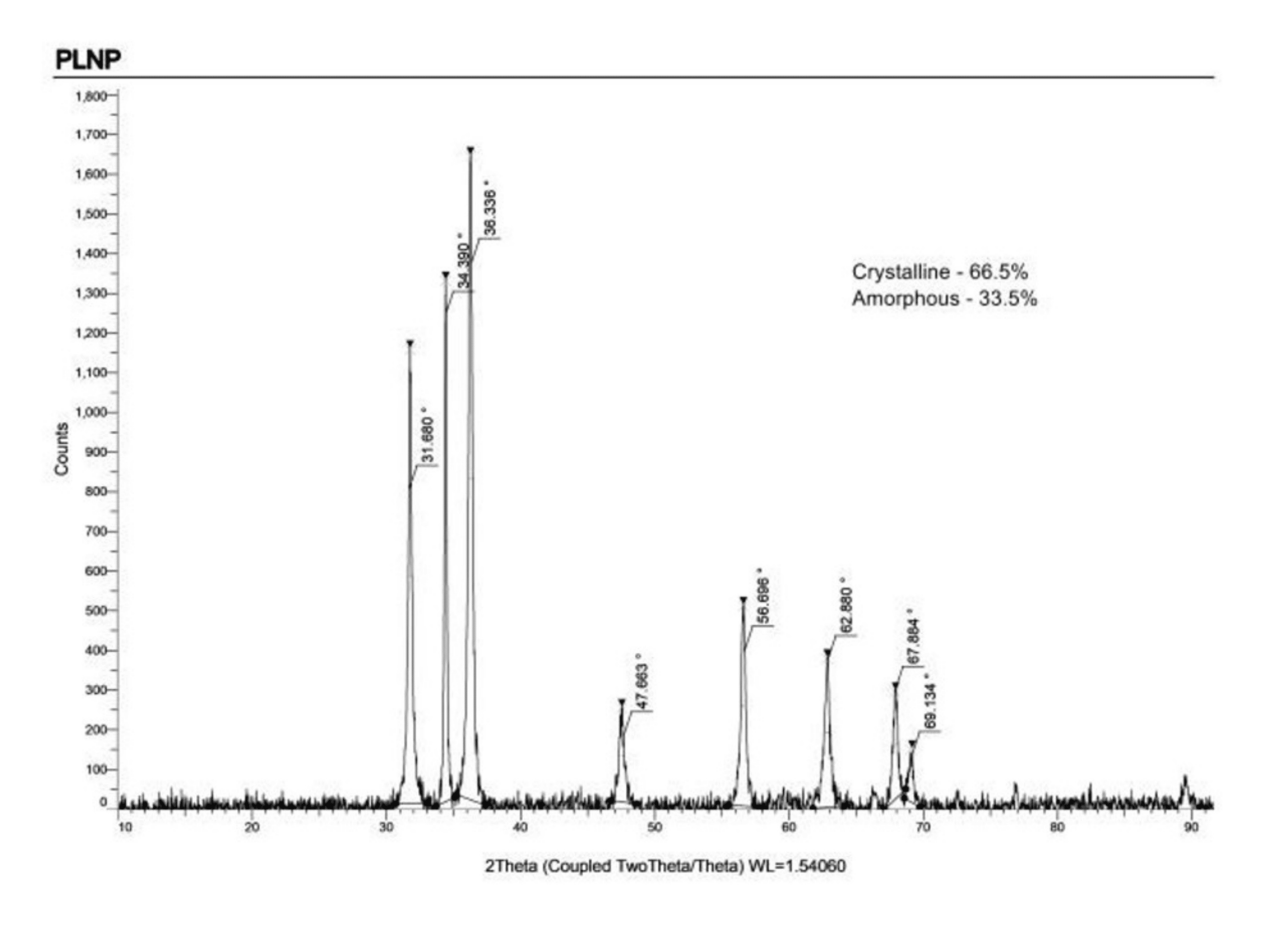
X-ray diffraction spectrum of PL ZnO NPs showed the crystalline peaks corresponding to nanoparticles.

The detection of ZnO NPs synthesized using *P. longum* leaf extract was established by XRD studies as depicted in Figure 3.

When the hexagonal structure of ZnO was evaluated using XRD, seven distinct diffraction peaks were found at 31.6°, 34.3°, 36.3°, 47.6°, 56.6°, 62.8°, 67.8, and 69.1°. These peaks were recorded at 800, 1250, 580, 1370, 190, 370, and 280 reflection planes, respectively (JCPDS No. 03-065-3411). For the strongest peak, d = 0.89/cos, the mean grain size generated during biosynthesis was determined to be 100 nm using the Scherrer formula. Sharp peaks and the lack of obscure peaks indicated that the produced nanoparticles were 66.5% pure and crystalline.

Scanning electron microscopy

**Figure 4:**
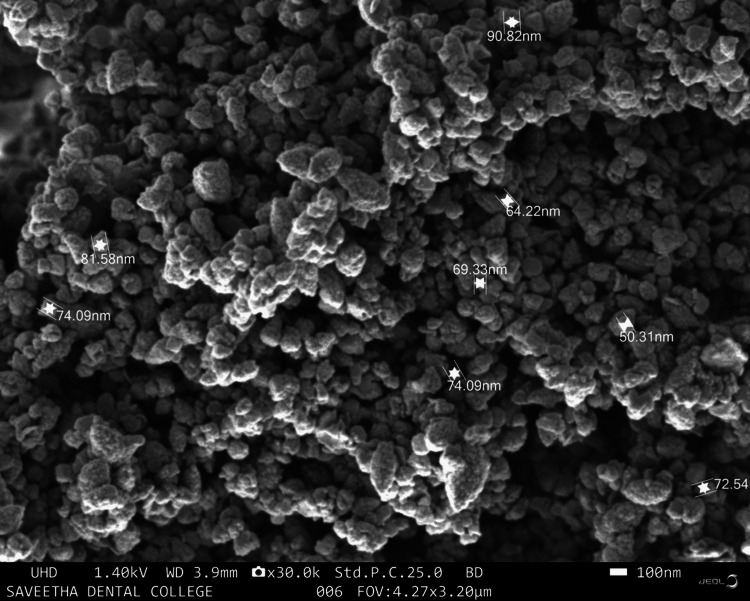
The scanning electron microscopic examination of the ZnO nanoparticles at 100x magnification.

Scanning electron micrographs were used to examine the surface morphology of ZnO NPs. A 200-nm-diameter SEM micrograph of ZnO NPs is displayed in Figure 4, and certain ZnO NPs possess a variety of morphologies, including irregular spheres, pentagons, and hexagons.

Microplastic degradation

**Figure 5:**
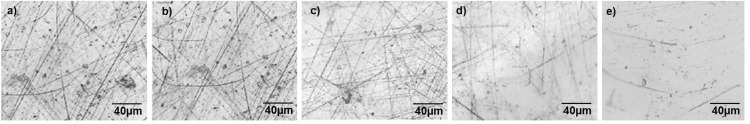
Pre-isolated dental microplastics degradation by PL ZnO NPs: (a) control without UV irradiation; (b) 15 μg/mL; (c) 50 μg/mL; (d) 75 μg/mL; and (e) 100 μg/mL.

The degradation efficacy of *P. longum* leaf extract-mediated zinc oxide nanoparticles (PL ZnO NPs) on pre-isolated dental microplastics was evaluated under varying concentrations and UV irradiation conditions. Figure 5 shows the results observed in the 100 μl concentration of ZnO NPs.

The samples were divided into five groups: a control without UV irradiation (control) and samples treated with PL ZnO NPs at concentrations of 15, 50, 75, and 100 μg/mL. The control group, which did not receive any UV irradiation, showed no significant degradation of dental microplastics, confirming that UV light is essential for activating the photocatalytic properties of PL ZnO NPs. At a concentration of 15 μg/mL, there was a minimal degradation of dental microplastics. This indicates that a lower concentration of PL ZnONPs is less effective in initiating the degradation process under UV light. Increasing the concentration to 50 μg/mL resulted in a noticeable improvement in microplastic degradation. The enhanced photocatalytic activity at this concentration suggests a positive correlation between PL ZnO NPs concentration and degradation efficiency. Further increasing the concentration to 75 μg/mL continued to show improved degradation of dental microplastics. The degradation observed was significantly higher than that at 50 μg/mL, indicating that higher concentrations of PL ZnO NPs enhance the photocatalytic breakdown of microplastics. The highest concentration of 100 μg/mL of the ZnO NPs demonstrated the most substantial degradation of dental microplastics among all the tested concentrations. This concentration achieved the highest efficiency, confirming that the degradation rate is maximum at this level. Overall, the results show that the degradation efficiency of dental microplastics increases with the concentration of PL ZnONPs, with the highest amount of degradation observed at the 100 μg/mL concentration. This study highlights the potential of PL ZnO NPs as an effective agent for the photocatalytic degradation of microplastics when activated by UV light. The highest amount of degradation was observed at 100 μl concentration of ZnO NPs. 

## Discussion

The synthesis and characterization of ZnO NPs using *P. longum* leaf extract revealed their structural and functional properties, as well as their potential application in environmental remediation, particularly in microplastic degradation [19]. The UV-Vis spectroscopy results indicated the successful synthesis of ZnO NPs, as evidenced by the distinct absorption peaks between 300 and 600 nm. This broad range of absorption is typical for ZnO NPs and suggests the formation of nanoparticles with varying sizes and shapes, which could influence their photocatalytic properties. The color change from pale yellowish-brown to dark brown further confirms the reduction of zinc ions and the formation of ZnO NPs. Similar results have been reported in other studies where plant extracts have been used for nanoparticle synthesis, indicating that the phytochemicals in *P. longum* play a crucial role in the reduction and stabilization of ZnO NPs [20].

FTIR analysis provided detailed information on the functional groups present in the synthesized ZnO NPs. The identification of O-H and N-H functional groups suggests that proteins and other phytochemicals in the *P. longum* extract may have acted as capping agents, stabilizing the nanoparticles during their formation [18]. The presence of these functional groups is essential for the stability and functionality of the nanoparticles, as they prevent agglomeration and enhance the biocompatibility of ZnO NPs. The findings align with previous studies where plant-based synthesis of nanoparticles has shown similar functional group participation, highlighting the importance of these biomolecules in the biosynthesis process. XRD analysis confirmed the crystalline nature of the synthesized ZnO NPs, with distinct diffraction peaks corresponding to the hexagonal structure of ZnO. The sharpness of the peaks and the absence of obscure peaks indicates high purity and crystallinity of PL ZnO NPs. The average grain size of 100 nm, calculated using Scherrer formula, suggests that the biosynthesized ZnO NPs are within the optimal size range for effective photocatalytic activity. This size-dependent property is crucial for the nanoparticles' ability to interact with and degrade microplastics under UV irradiation, as smaller NPs provide a larger surface area for the catalytic reactions [14].

The SEM images showed the diverse morphology of the ZnO NPs, with particles exhibiting irregular spherical, pentagonal, and hexagonal shapes. The variation in morphology could be attributed to the different phytochemicals in the *P. longum* extract, which may influence the nucleation and growth of the nanoparticles [12]. The diverse morphology of ZnO NPs is advantageous for their application in environmental remediation, as it can affect the surface area and, consequently, the photocatalytic efficiency of the nanoparticles. The microplastic degradation study demonstrated that the photocatalytic activity of *P. longum*-mediated ZnO NPs (PL ZnO NPs) is concentration-dependent.

The current strategies for managing waste plastics encompass photo-oxidative, thermal, ozonation, catalytic, mechanical, and chemical degradation methods [21]. Nonetheless, research indicates that photocatalysis may offer a promising, low-cost, and energy-efficient solution for polymer degradation. This process entails the use of light to stimulate nanostructured semiconductors, resulting in the formation of exciton pairs that interact with the surrounding water to generate highly reactive species such as hydroxyl and superoxide radicals, which are capable of effectively oxidizing and breaking down polymers [22,23]. Previous literature has demonstrated that titanium oxide nanoparticles and nanoflowers effectively degrade microplastics [22- 26]. The photocatalytic degradation of zinc nanoparticles (ZnO NPs) has been studied in relation to microplastics derived from aquatic environments, demonstrating the successful degradation of microplastics derived from saliva [27].

In the present study, the degradation efficiency increased with the concentration of PL ZnO NPs, with the highest degradation observed at 100 μg/mL under UV irradiation [26]. This finding is significant as it underscores the potential of PL ZnO NPs in the degradation of dental microplastics, which are persistent pollutants in the environment [28]. The results suggest that higher concentrations of ZnO NPs provide more active sites for the photocatalytic reactions, leading to enhanced breakdown of microplastics. Previous studies have also reported the effectiveness of ZnO NPs in degrading various organic pollutants, further supporting the findings of this study.

The concentration-dependent degradation of microplastics under UV irradiation indicates the potential of these nanoparticles in environmental applications. The use of ZnO NPs for microplastic degradation shows promise. At low concentrations, ZnO NPs could be added to mouthwashes and food hygiene products to help reduce microplastic contamination due to their antimicrobial and oxidative properties. At higher concentrations, they may be useful in managing dental plastic waste, which is challenging to degrade. ZnO NPs could accelerate the breakdown of durable polymers in dental plastics, making waste management more efficient. Further research is needed to optimize ZnO NP concentrations and exposure times to maximize their degradation efficiency and explore their potential in both consumer products and industrial waste management.

Limitations

However, a limitation of the green synthesis method is its uncertain scalability. This is because translating lab-scale processes to industrial levels may present challenges in maintaining consistency and efficiency. The microplastic degradation at the lab scale showed a good degradation efficiency, but scaling up the microplastics degradation procedure to the plastic and waste management plant level is a different scenario and necessitates future studies to determine optimal nanoparticle concentration and degradation protocol. Future studies could explore the scalability of this synthesis method and the long-term environmental impact of using ZnO NPs for microplastic degradation.

## Conclusions

ZnO NPs were synthesized using *P. longum* leaf extract (PL ZnO NPs) and the formation of nanoparticles was confirmed through characterization studies such as FTIR, XRD, and SEM. Also, these nanoparticles were employed for the degradation of dental microplastics and the most effective degradation was observed at a concentration of 100 μg/mL under UV irradiation as a catalyst, whereas 75 μg/mL is the optimal concentration at which an extensive degradation of microplastics takes place.
